# Discussion on the duration of response following HBsAg clearance in patients with chronic hepatitis B treated with PegIFNα-2b

**DOI:** 10.3389/fimmu.2025.1518048

**Published:** 2025-04-08

**Authors:** Tao Wang, Fei Tang, Fenghui Li, Jing Chen, Fei Yan, Qin Du, Weili Yin, Jing Liang, Lei Liu, Fang Wang, Baiguo Xu, Qing Ye, Huiling Xiang

**Affiliations:** ^1^ Department of Gastroenterology and Hepatology, Tianjin University Central Hospital (Tianjin Third Central Hospital), Tianjin Key Laboratory of Extracorporeal Life Support for Critical Diseases, Tianjin Institute of Hepatobiliary Disease, Tianjin, China; ^2^ The Third Central Clinical College of Tianjin Medical University, Tianjin University Central Hospital (Tianjin Third Central Hospital), Tianjin Key Laboratory of Extracorporeal Life Support for Critical Diseases, Tianjin Institute of Hepatobiliary Disease, Tianjin, China; ^3^ Nankai University Affiliated Third Center Hospital, Tianjin University Central Hospital (Tianjin Third Central Hospital), Tianjin Key Laboratory of Extracorporeal Life Support for Critical Diseases, Tianjin Institute of Hepatobiliary Disease, Tianjin, China

**Keywords:** chronic hepatitis B, pegylated interferon alpha-2b, functional cure, HBsAg clearance, HBsAg reversion, sustained response

## Abstract

**Aim:**

Functional cure strategies based on interferon therapy for chronic hepatitis B (CHB) are gaining increasing attention among clinicians. However, studies investigating the duration of response after achieving HBsAg clearance with interferon treatment are limited. This study aims to explore the patterns of sustained response following HBsAg clearance in patients treated with pegylated interferon alpha-2b (PegIFNα-2b) through long-term follow-up, providing guidance for clinical practice.

**Methods:**

We collected data from CHB patients who achieved HBsAg clearance and were treated with either PegIFNα-2b monotherapy or in combination with nucleos(t)ide analogs (NAs) at Tianjin Third Central Hospital from January 2018 to May 2024. Regular follow-up assessments were conducted to observe the dynamic changes in HBsAg, HBV DNA, and liver function during the follow-up period. We recorded the time to HBsAg reversion (defined as HBsAg ≥ 0.05 IU/mL), analyzed the patterns of HBsAg reversion, and investigated the optimal time points for evaluating sustained HBsAg clearance.

**Results:**

A total of 173 patients with CHB or compensated hepatitis B cirrhosis were included. The mean age was 41.5 ± 9.0 years, with 16.19% of patients having compensated cirrhosis. The median follow-up duration was 89.3 weeks (range: 18.6 to 289.1 weeks). HBsAg reversion occurred in 26 patients, yielding a reversion rate of 15.03% (26/173). Among these 26 patients, 50% (13/26) experienced reversion within 24 weeks, and 80.77% within 48 weeks; thereafter, the number of reversions gradually decreased. At 48 weeks post-treatment cessation, the HBsAg sustained response rate was 95.45%, stabilizing at 100% after 120 weeks. Among patients with regular follow-ups, virtually none experienced reversion beyond 72 weeks. At the time of HBsAg reversion, all 26 patients exhibited normal alanine aminotransferase (ALT), aspartate aminotransferase (AST), and total bilirubin (TBIL) levels, with a median HBsAg level of 0.70 IU/mL (range: 0.05 to 8.13 IU/mL), and only one patient showing low-level positive HBV DNA (117 IU/mL). No adverse events, including liver failure, decompensation, or hepatocellular carcinoma, occurred during the follow-up period.

**Conclusions:**

Patients with chronic hepatitis B treated with PegIFNα-2b demonstrated favorable long-term persistence of HBsAg clearance. However, there remains a risk of HBsAg reversion after treatment cessation, predominantly within the first 48 weeks. HBsAg sustained response (HSR) at 48 weeks post-treatment is a critical follow-up time point for CHB patients post-HBsAg clearance, with HSR at 72 weeks potentially representing an ideal follow-up timeframe, while HSR at 120 weeks may serve as a marker for extended follow-up.

## 1 Introduction

Chronic hepatitis B (CHB) is a progressive liver disease associated with significant morbidity, including cirrhosis and hepatocellular carcinoma (HCC) (1). Hepatitis B surface antigen (HBsAg) seroclearance is a critical therapeutic endpoint, as it correlates with improved liver function, histological recovery, and enhanced long-term clinical outcomes. Consequently, HBsAg clearance has been established as an optimal treatment goal in major international and domestic guidelines (2–7). However, spontaneous HBsAg clearance occurs rarely, and even prolonged nucleos(t)ide analog (NA) therapy achieves only minimal clearance rates (0–3%) (8, 9). Recent advances in therapeutic strategies incorporating pegylated interferon-alpha (PegIFN-α) have demonstrated markedly higher HBsAg clearance rates in CHB patients, driving its growing adoption in clinical practice. Despite these advancements, the durability of virological remission after HBsAg loss remains poorly characterized. This study aims to investigate the long-term trajectories of virological response in CHB patients following PegIFN-α-induced HBsAg clearance. By identifying optimal end points for assessing sustained response, our findings seek to inform evidence-based strategies for post-treatment monitoring and functional cure management.

## 2 Study design and methods

### 2.1 Study participants

This study included patients with CHB and compensated hepatitis B cirrhosis who visited the Gastroenterology and Hepatology Department at Tianjin Third Central Hospital from January 2018 to May 2024. The diagnostic criteria were based on the Chinese Guidelines for the Prevention and Treatment of Chronic Hepatitis B (2022 Edition) (7).

Inclusion criteria were as follows: (1) HBsAg positivity for at least 6 months; (2) treatment with PegIFNα-2b (Pegbin^®^, Xiamen Amoytop Biotech Co., Ltd.) alone or in combination with NAs for over 24 weeks; (3) achievement of HBsAg clearance during treatment; (4) regular follow-up every 3 to 6 months post-clearance.

Exclusion criteria included: (1) co-infection with other viruses (e.g., human immunodeficiency virus [HIV], hepatitis D virus [HDV], hepatitis C virus [HCV]), liver cancer, alcoholic liver disease, autoimmune hepatitis, or other liver diseases; (2) decompensated cirrhosis, including a history of complications such as gastrointestinal bleeding, ascites, or hepatic encephalopathy; (3) pregnant or breastfeeding women; (4) patients using corticosteroids, immunosuppressants, immunotherapy drugs, or those with a history of alcohol or drug abuse during the follow-up period; (5) patients who had not discontinued interferon treatment; (6) patients who did not adhere to regular follow-up. The initial dose of Peg-IFN α-2b for patients was 180 micrograms (μg), administered once weekly. During the course of treatment, dose adjustments were made according to the instructions. Dose reduction should be considered, if absolute neutrophil count (ANC) < 0.75 × 10^9^/L or PLT < 50 × 10^9^/L. Discontinuation of PegIFN was necessary if ANC < 0.50 × 10^9^/L, PLT < 25 × 10^9^/L or serious adverse events (AEs) occurred. The diagnostic criteria for MAFLD (metabolic-associated fatty liver disease) refer to the international expert consensus statement (10).

HBsAg clearance was defined as a serological conversion to negative HBsAg (HBsAg < 0.05 IU/mL) following PegIFNα-2b treatment. HBsAg sustained response was defined as sustained HBsAg loss with or without the appearance of HBsAb at the end of follow-up (EOF). HBsAg reversion was defined as the re-detection of positive HBsAg (≥ 0.05 IU/mL) during follow-up after an initial conversion to negative.

### 2.2 Study design

Patients were categorized into two groups based on the presence of HBsAg (≥0.05 IU/mL) during follow-up after discontinuation of PegIFNα-2b treatment: the HBsAg sustained response (HSR) group and the HBsAg reversion (HRV) group. For patients in the HRV group, the study endpoint was defined as the first detection of HBsAg reversion during follow-up, while HBsAg-negative patients continued follow-up until the conclusion of the study. Consolidation therapy (weeks) refers to the period of continued interferon treatment after HBsAg clearance; whereas, the therapy duration (weeks) represents the total time from the initiation to the cessation of interferon treatment.

### 2.3 Clinical indicators assessment

Patient demographic information (including age and sex) was recorded along with baseline peripheral blood liver function tests, complete blood counts, hepatitis B serological markers including HBV DNA, quantitative HBsAg levels, the date of first HBsAg clearance, date of HBsAg reversion, types of NAs used, details of interferon treatment, and adverse reactions. After HBsAg clearance, patients were followed up approximately every 3 months, during which hepatitis B virus markers, biochemical indicators, and clinical adverse events were documented.

Quantitative HBV DNA Detection: HBV DNA was measured using the Roche COBAS AmpliPrep/COBAS TaqMan HBV Test v2.0, with a detection range of 20 to 1.70 × 10^8^ IU/mL.

Quantitative HBsAg Detection: Quantitative detection of HBsAg was performed using the Abbott ARCHITECT i4000SR chemiluminescent microparticle immunoassay (Abbott Diagnostics, Abbott Park, IL, USA), with a detection range of 0.05 to 250 IU/mL. Samples with concentrations >250 IU/mL were diluted for quantitative analysis. Levels of HBsAb and HBeAg were determined using commercial immunoassay kits.

### 2.4 Statistical methods

For normally distributed continuous variables, results are expressed as mean ± standard deviation $\left(\bar{x}\pm s\right)$ , with comparisons between groups using the t-test. Categorical data were compared using the chi-square test and trend chi-square test. For non-normally distributed continuous variables, data are presented as median (min-max) and group comparisons were conducted using the Mann-Whitney U test. A p-value of <0.05 was considered statistically significant. All statistical analyses were conducted using the R statistical software package (http://www.R-project.org, The R Foundation) and EmpoweringStats (http://www.empowerstats.com, X&Y Solutions, Inc.). Graphs were created using Origin 2022b software.

### 2.5 Ethics

This study was conducted in accordance with the ethical principles outlined in the Declaration of Helsinki and was approved by the Ethics Committee of Tianjin Third Central Hospital (Approval No: IRB2020-015-01). All data analyses were performed under the principle of anonymity. As a real-world cohort study, no additional specimen collection was conducted due to the study, and it did not affect patients’ treatment plans, disease progression, or prognosis; therefore, it met the criteria for a waiver of informed consent.

## 3 Results

### 3.1 Demographic and baseline characteristics

A total of 195 patients were enrolled in this study. Twenty patients who were still undergoing treatment and two who were lost to follow-up were excluded, resulting in a final cohort of 173 patients. The mean age of participants was 41.5 ± 9.0 years, with 117 males (67.63%), including 28 patients with compensated cirrhosis (16.19%) and 89 patients with concurrent MAFLD (51.445%). Among the 173 patients, 147 maintained HBsAg negativity throughout the follow-up period (HSR group), yielding a sustained HBsAg response rate of 84.97% (147/173). In contrast, 26 patients were found to have at least one positive HBsAg during follow-up (HRV group), resulting in an overall HBsAg reversion rate of 15.03% (26/173). The median duration of therapy for patients in the HRV group was 58.00 weeks (with a range of 42.50 to 71.25 weeks), whereas for patients in the HSR group, it was 48.00 weeks (ranging from 30.00 to 62.00 weeks). The difference between the two groups was not statistically significant. Additionally, no significant differences were observed between the two groups regarding age, types of NAs used, prior treatment history, treatment regimens, presence of MAFLD, continuation of NAs therapy after the cessation of interferon, baseline alanine aminotransferase (ALT), aspartate aminotransferase (AST), platelets (PLT), HBV DNA levels or baseline HBeAg status before interferon treatment. However, the proportion of males and baseline HBsAg levels were significantly higher in the HRV group compared to the HSR group. Additionally, there were statistically significant differences between the two groups in terms of baseline HBsAg, HBsAb level at the end of treatment and whether the consolidation therapy (weeks) ≥12 weeks (*p < 0.05*) (**Table 1**).

**Table 1:** Comparison of baseline characteristics between HSR and HRV groups.

	Total	HSR	HRV	*P*
**N (%)**	173	147 (84.97)	26 (15.03)	
**Age** (years) (Mean ± SD)	41.509 ± 9.022	41.646 ± 9.265	40.731 ± 7.613	0.682
**Sex**				**0.045**
Male (n, %)	117 (67.630%)	95 (64.626%)	22 (84.615%)	
Female (n, %)	56 (32.370%)	52 (35.374%)	4 (15.385%)	
**Combined NAs**				0.365
No (n, %)	52 (30.058%)	48 (32.653%)	4 (15.385%)	
ETV (n, %)	53 (30.636%)	43 (29.252%)	10 (38.462%)	
TDF (n, %)	46 (26.590%)	38 (25.850%)	8 (30.769%)	
TAF (n, %)	22 (12.717%)	18 (12.245%)	4 (15.385%)	
**Treat history**				0.322
Naive (n, %)	76 (43.931%)	68 (46.259%)	8 (30.769%)	
NAs treated (n, %)	78 (45.087%)	64 (43.537%)	14 (53.846%)	
IFN treated (n, %)	19 (10.983%)	15 (10.204%)	4 (15.385%)	
**Treat regimens**				0.0588
IFN (n, %)	54 (31.214%)	50 (34.014%)	4 (15.385%)	
NAs+IFN (n, %)	119 (68.786%)	97 (65.986%)	22 (84.615%)	
**Cirrhosis**				0.1068
No	145 (83.815%)	126 (85.714%)	19 (73.077%)	
Yes	28 (16.185%)	21 (14.286%)	7 (26.923%)	
**MAFLD**				0.4893
No	84 (48.555%)	73 (49.660%)	11 (42.308%)	
Yes	89 (51.445%)	74 (50.340%)	15 (57.692%)	
**ALT** (U/L) [Median(IQR)]	22.500 (15.000-37.750)	23.000 (14.750-40.250)	22.000 (19.000-33.250)	0.848
**AST** (U/L) [Median(IQR)]	21.500 (18.000-28.250)	21.000 (18.000-29.250)	22.000 (17.425-25.250)	0.619
**PLT** (×10^9/L) (Mean ± SD)	210.869 ± 75.367	213.155 ± 72.436	198.149 ± 90.774	0.583
**HBsAg** (IU/ml, lg) (Mean ± SD)	1.586 ± 1.355	1.481 ± 1.353	2.175 ± 1.229	**0.009**
**HBV DNA** (IU/ml, lg) [Median(IQR)]	0.000 (0.000-1.908)	0.000 (0.000-1.979)	0.000 (0.000-1.710)	0.867
**HBsAb** (IU/ml) [Median(IQR)]	114.080 (19.690-257.252)	127.550 (23.240-280.670)	47.840 (16.032-131.495)	**0.014**
**HBeAg**				0.658
Negtive (n, %)	151(87.283%)	129 (87.755%)	22 (84.615%)	
positive (n, %)	22 (12.717%)	18 (12.245%)	4 (15.385%)	
**Continue NAs**				0.6405
No	106 (61.272%)	89 (60.544%)	17(65.385%)	
Yes	67 (38.728%)	58 (39.456%)	9(34.615%)	
**Consolidation therapy** (weeks) [Median(IQR)]	12.000 (4.000-12.000)	12.000 (8.000-13.500)	7.000 (0.000-12.000)	**0.023**
**Consolidation therapy** (weeks)≥12				**0.029**
No	79 (45.665%)	62 (42.177%)	17 (65.385%)	
Yes	94 (54.335%)	85 (57.823%)	9 (34.615%)	
**Therapy duration** (weeks) [Median(IQR)]	48.000 (30.000-71.250)	48.000 (30.000-62.000)	58.000 (42.500-71.250)	0.053

SD, standard deviation; IQR: Interquartile Range;NAs, Nucleoside Analogs; ETV, Entecavir; TDF, Tenofovir disoproxil fumarate; TAF, Tenofovir alafenamide fumarate tablets; MAFLD, metabolic-associated fatty liver disease; ALT, Alanine aminotransferase; AST, Aspartate Aminotransferase; PLT, platelet; HBsAg, hepatitis B surface antigen; HBsAb, hepatitis B surface antibody; HBeAg, hepatitis B envelope antigen; HBV DNA, hepatitis B virus deoxyribonucleic acid; Continue NAs: whether continue NAs after the cessation of interferon. The values marked in bold signify statistical significance (p<0.05).

### 3.2 Risk factors for HBsAg reversion

Age, gender, types of combined NAs, prior treatment history, treatment regimen, presence of cirrhosis, presence of MAFLD, baseline liver function, HBsAg, HBV DNA, baseline HBeAg status, HBsAb levels at the end of treatment, continuation of NAs treatment after treatment completion, and consolidation therapy (weeks) ≥12 weeks, therapy duration (weeks) were used as independent variables to establish the Cox proportional risk model. The univariate analysis results indicated a significant association between HBsAg reversion and baseline HBsAg, HBsAb at the end of treatment and consolidation therapy (weeks) ≥12 weeks. Multivariate analysis results showed that a high level of HBsAb at the end of treatment was a protective factor, while baseline HBsAg was risk factor for HBsAg reversion (*p < 0.05*) (**Table 2**).

**Table 2:** Cox regression analysis of factors associated with HBsAg reversion.

	Statistics	Univariable(*HR, 95% CI, Pvalue*)	Multivariable(*HR, 95% CI, Pvalue*)
**Age** (**years)** (**Mean** ± **SD)**	41.509 ± 9.022	0.995 (0.953, 1.039) 0.81982	
Gender			
Male (n, %)	117 (67.630%)	1	
Female (n, %)	56 (32.370%)	0.383 (0.132, 1.111) 0.07738	
Combined NAs			
No (n, %)	52 (30.058%)	1	
ETV (n, %)	53 (30.636%)	1.973 (0.617, 6.313) 0.25186	
TDF (n, %)	46 (26.590%)	2.128 (0.640, 7.077) 0.21811	
TAF (n, %)	22 (12.717%)	3.791 (0.938, 15.324) 0.06150	
Treat history			
Naiive (n, %)	76 (43.931%)	1	
NAs treated (n, %)	78 (45.087%)	1.440 (0.603, 3.439) 0.41141	
IFN treated (n, %)	19 (10.983%)	1.795 (0.539, 5.975) 0.34011	
Treat regimens			
IFN (n, %)	54 (31.214%)	1	
NAs+IFN (n, %)	119 (68.786%)	2.410 (0.830, 7.000) 0.10588	
Cirrhosis			
NO	145 (83.815%)	1	
Yes	28 (16.185%)	2.126 (0.888, 5.088) 0.09044	
MAFLD			
NO	84 (48.555%)	1	
Yes	89 (51.445%)	1.342 (0.616, 2.927) 0.45919	
**ALT** (IU/ml)	45.217 ± 81.600	0.997 (0.989, 1.006) 0.50856	
**AST** (IU/m)	31.639 ± 37.800	0.993 (0.975, 1.011) 0.44092	
**PLT** (×10^9/L)	210.869 ± 75.367	0.997 (0.992, 1.003) 0.37903	
**HBsAg** (IU/ml, lg)	1.586 ± 1.355	1.422 (1.042, 1.942) 0.02653	1.519 (1.058, 2.180) 0.02353
**HBVDNA**(IU/ml, lg)	1.071 ± 1.925	0.996 (0.813, 1.220) 0.96557	
**HBsAb** (IU/ml)	201.140 ± 262.708	0.996 (0.993, 0.999) 0.01377	0.996 (0.993, 0.999) 0.01310
HBeAg			
Negtive	147 (90.741%)	1	
Positive	15 (9.259%)	2.157 (0.728, 6.395) 0.16558	
Continue NAs			
NO	106 (61.628%)	1	
Yes	66 (38.372%)	0.875 (0.390, 1.963) 0.74592	
**Consolidation therapy**(weeks)	11.457 ± 12.045	0.985 (0.961, 1.009) 0.20681	
Consolidation therapy(weeks)≥12			
NO	79 (45.665%)	1	
Yes	94 (54.335%)	0.436 (0.194, 0.978) 0.04395	0.590 (0.260, 1.339) 0.20683
**Therapy duration** (weeks)	52.867 ± 33.227	1.004(0.996, 1.013) 0.32648	

CI, confidence interval; HR, hazard ratio; NAs, Nucleoside Analogs; ETV, Entecavir; TDF, Tenofovir disoproxil fumarate; TAF, Tenofovir alafenamide fumarate tablets; IFN, interferon; MAFLD, metabolic-associated fatty liver disease; ALT, Alanine aminotransferase; AST, Aspartate Aminotransferase; PLT, platelet; HBsAg, hepatitis B surface antigen; HBsAb, hepatitis B surface antibody; HBeAg, hepatitis B envelope antigen; HBV DNA, hepatitis B virus deoxyribonucleic acid; Continue NAs: whether continue NAs after the cessation of interferon.

### 3.3 Follow-up of patients with HBsAg clearance

From the date of initial HBsAg clearance to the conclusion of the study (August 1, 2024), the follow-up duration ranged from a minimum of 18.6 weeks to a maximum of 289.1 weeks, with a mean follow-up of 96.42 ± 54.1 weeks and a median follow-up of 89.3 weeks (range: 18.6 to 289.1 weeks). The follow-up duration from the cessation of interferon treatment to the conclusion of the study varied from 0 weeks to 277.0 weeks, with a mean of 81.64 ± 51.99 weeks and a median of 73.3 weeks (range: 0.0 to 277.0 weeks).

The average time to HBsAg reversion for the HRV group was 41.71 ± 27.02 weeks after the initial HBsAg clearance and 30.94 ± 25.08 weeks after discontinuation of interferon treatment, with median times of 37.7 weeks and 25.95 weeks, respectively.

### 3.4 Distribution of HBsAg reversion time

**Figure 1:**
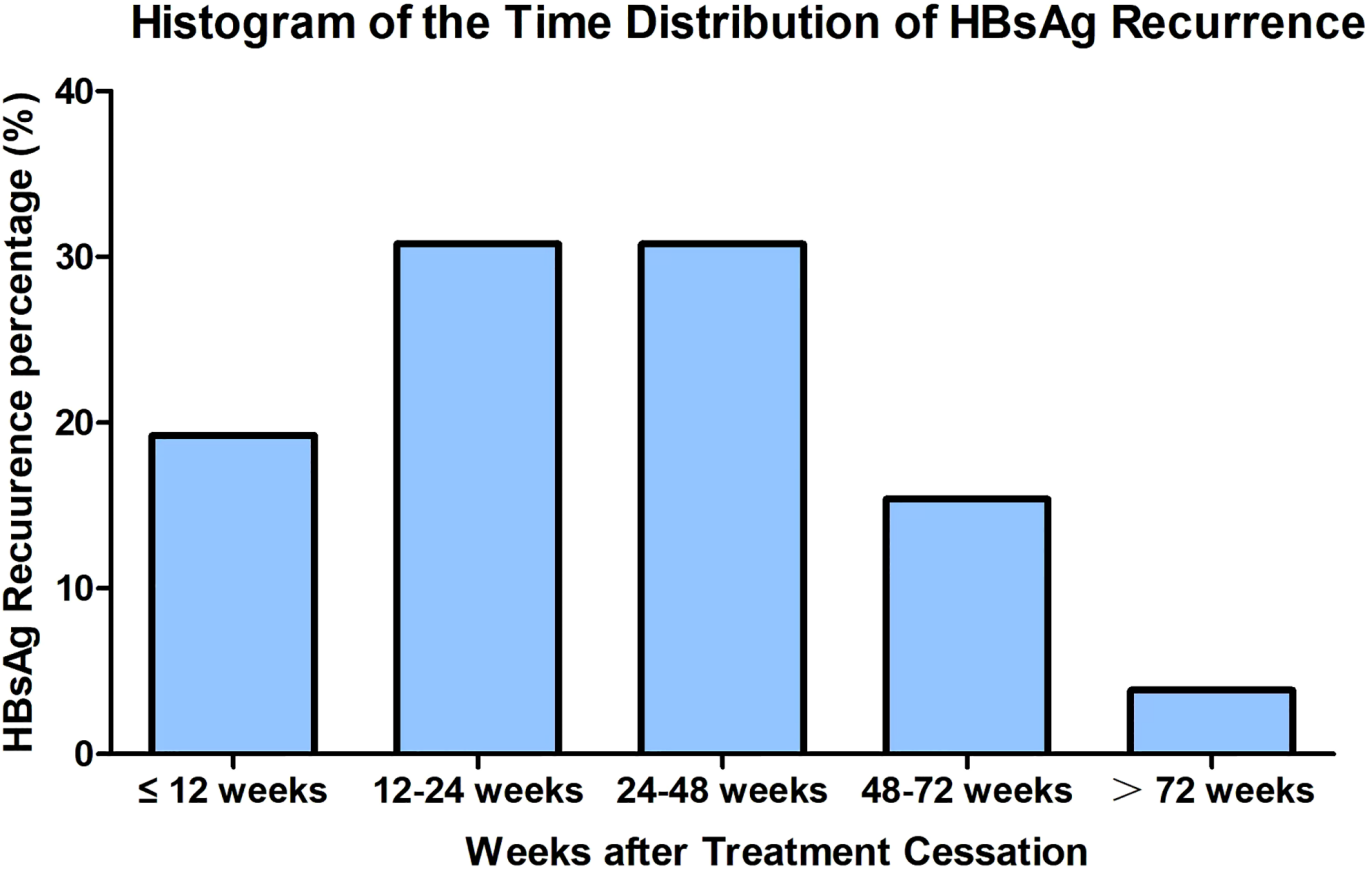
Histogram of the time distribution of HBsAg recurrence.

Among the 26 patients with HBsAg reversion, five patients (19.23%) experienced reversion within 12 weeks after stopping PegIFNα-2b treatment. Eight patients (30.77%) had reversion between 12 to 24 weeks, and another eight patients (30.77%) between 24 to 48 weeks. Four patients (15.38%) experienced reversion between 48 to 72 weeks, while one patient (3.85%) had reversion after 72 weeks. Notably, 50% (13/26) of HBsAg reversion occurred within 24 weeks, and 80.77% occurred within 48 weeks, with the rate of HBsAg reversion gradually decreasing beyond 48 weeks (**Table 3**, **Figure 1**). We grouped patients based on whether their HBsAg levels were above 500 IU/mL and whether their HBsAb levels were above 100 IU/mL, respectively, and found no statistically significant difference in the distribution of HBsAg reversion time among the groups (*p > 0.05*) (**Supplementary Table S1**).

**Table 3:** Distribution of HBsAg Reversion Time.

Time (weeks)	HBsAg Reversion (n)	Percentage (%)	Cumulative Reversions (n)	Cumulative Percentage (%)
≤12 weeks	5	19.23%	5	19.23%
12-24 weeks	8	30.77%	13	50.00%
24-48 weeks	8	30.77%	21	80.77%
48-72 weeks	4	15.38%	25	96.15%
>72 weeks	1	3.85%	26	100.00%
	26	100%		

### 3.5 Analysis of HSR rate following HBsAg clearance

During the entire follow-up period after discontinuation of PegIFNα-2b treatment, a high sustained response rate was observed after HBsAg clearance, which increased progressively with the duration of observation. At 48 weeks post-treatment cessation, the HSR rate reached 95.45%, and this rate stabilized at 100% at 120 weeks.

**Figure 2:**
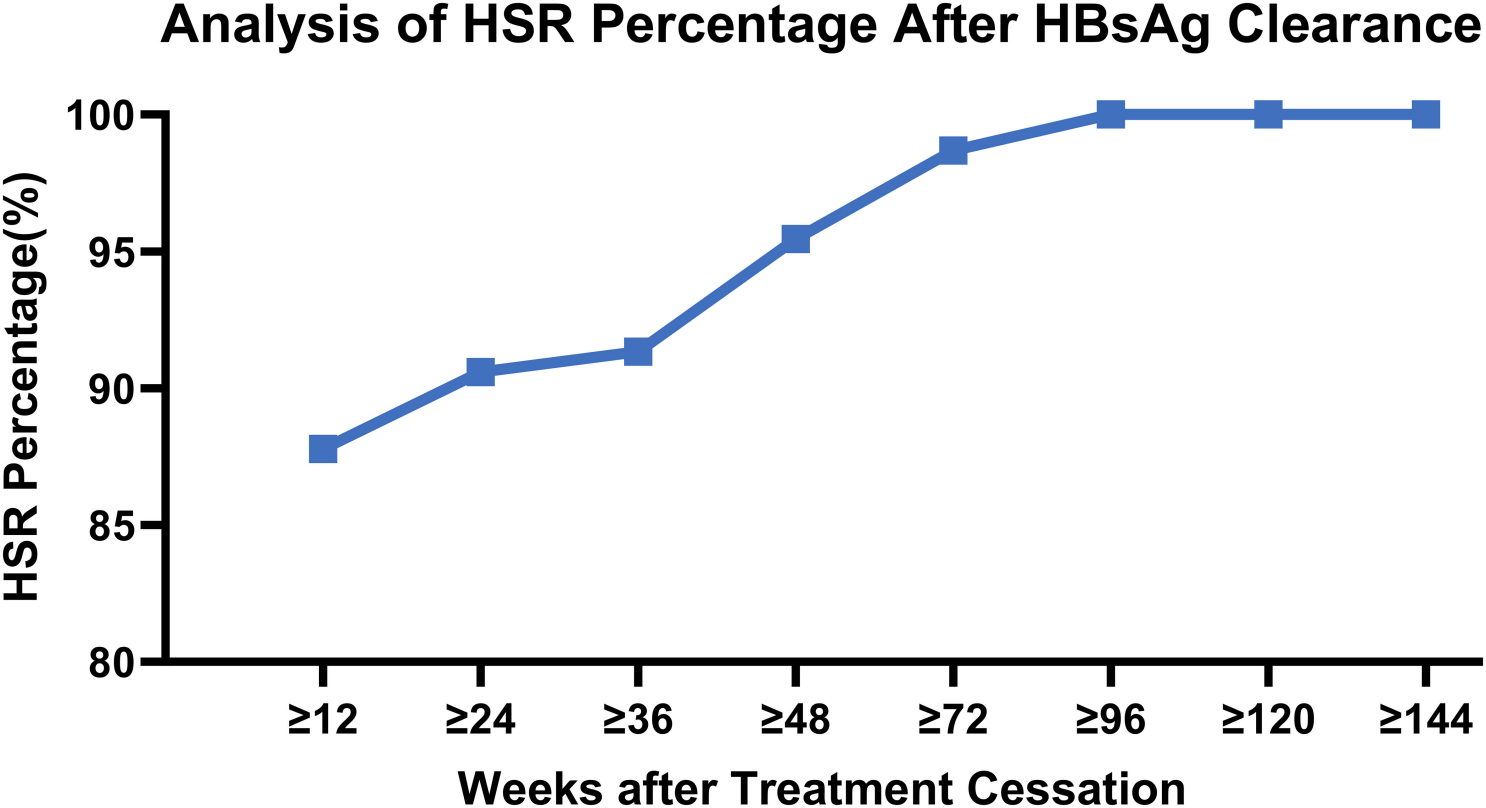
Analysis of HSR percentage after HBsAg clearance.

Among the patients with HBsAg reversion, the latest occurrence was noted at 107.3 weeks following the cessation of PegIFNα-2b; however, this patient had not been followed up for 79 weeks prior to reversion. All cases of HBsAg reversion occurred within 72 weeks, except for this one, and no new reversion events were observed after this period. (**Table 4**, **Figure 2**).

**Table 4:** Analysis of HSR rate following HBsAg clearance.

	HSR (n)	HRV (n)	Total (n)	HSR rate
**Total**	147	26	173	84.97%
**≥12**	151	21	172	87.79%
**≥24**	135	14	149	90.60%
**≥36**	116	11	127	91.34%
**≥48**	105	5	110	95.45%
**≥72**	75	1	76	98.68%
**≥96**	44	0	44	100.00%
**≥120**	21	0	21	100.00%
**≥144**	13	0	13	100.00%

Thus, patients who achieve HBsAg clearance following PegIFNα-2b treatment exhibit favorable long-term persistence, with most reversions occurring within 48 weeks post-treatment, and nearly no patients experiencing reversion after 72 weeks, resulting in a 0% reversion rate after 120 weeks. Clinically, HSR at 48 weeks may serve as a critical follow-up time point following interferon therapy, with HSR at 72 weeks potentially representing an ideal follow-up interval, and HSR at 120 weeks serving as a marker for extended follow-up duration.

### 3.6 Safety analysis at the time of HBsAg reversion and follow-up

Among the 26 patients who experienced HBsAg reversion, the median levels at the time of reversion were as follows: ALT 40.01 U/L (range: 25.02 U/L - 58.00 U/L), AST 19.00 U/L (range: 6.00 U/L - 48.00 U/L), total bilirubin (TBIL) 13.80 μmol/L (range: 5.70 μmol/L - 22.00 μmol/L). Of the 26 patients with HBsAg reversion, only one had a concurrent positive HBV DNA result (117 IU/mL), with a negative HBeAg status, while 96.1% (25/26) maintained HBV DNA negativity. Throughout the treatment and follow-up periods, no adverse events such as liver failure, decompensated cirrhosis, or hepatocellular carcinoma occurred in any patient (detailed information is presented in **Table 5**).

**Table 5:** Detailed information of patients with HBsAg reversion.

No	Sex	Age	HBsAg^a^	HBeAg ^a^	HBeAg ^b^	HBeAg ^c^	HBsAg ^b^	HBV DNA	ALT	AST	TBIL	Presence of CR	Continue NAs	HBsAb	Vaccination Status
1	1	40	0.41	0	0	0	55.04	0	15	18	18.6	1	1	20.68	1
2	1	40	0.05	0	0	0	907.61	0	48	44	10.4	0	1	15.11	1
3	1	58	0.05	0	0	0	1174.90	0	25	29	11.2	1	1	0	0
4	2	40	0.06	0	0	0	2969.71	0	9	20	12.2	0	1	154.48	0
5	1	36	0.41	0	0	0	338.49	0	15	26	15.9	0	1	561.11	1
6	1	45	0.07	0	0	0	402.98	0	36	37	12.4	0	0	0.88	0
7	2	54	0.49	0	0	0	479.31	0	10	19	14.8	0	0	90.68	0
8	1	37	0.89	1	0	0	731.26	1	6	18	22	0	1	230.52	0
9	1	47	0.75	0	0	0	1043.58	0	17	18	16	1	1	296.59	1
10	1	30	0.15	0	0	0	0.16	0	11	13	17.5	0	0	0.57	NA
11	1	34	0.09	1	0	0	14355.01	0	29	17	15.8	0	1	0.83	NA
12	1	36	0.29	0	0	0	267.21	0	23	26	8.5	0	1	80.21	1
13	2	25	0.17	0	0	0	769.95	0	22	27	12.4	0	1	52.83	1
14	2	32	0.17	0	0	0	0.20	0	10	16	5.7	0	1	51.98	0
15	1	42	0.28	1	0	0	126.12	0	36	113	12.8	0	1	0	0
16	1	39	0.97	0	0	0	10.76	0	10	17	8.5	0	0	18.8	1
17	1	47	0.18	0	0	0	2.65	0	16	21	20.3	0	0	8.69	1
18	1	37	8.13	0	0	0	410.51	0	17	19	16.8	0	0	211	1
19	1	44	0.57	0	0	0	67.12	0	37	25	10.1	0	0	145.1	1
20	1	41	0.34	0	0	0	2885.99	0	22	26	17.6	1	1	165.1	1
21	1	39	0.15	1	0	0	471.14	0	17	25	11.8	0	1	26.59	0
22	1	52	0.37	0	0	0	466.71	0	23	22	15.6	1	1	19.97	NA
23	1	30	2.44	0	0	0	14.29	0	20	16	16.1	0	0	43.7	0
24	1	45	0.37	0	0	0	2.89	0	18	22	9.4	1	1	22.3	1
25	1	42	0.05	0	0	0	364.60	0	47	32	12.6	0	1	53.38	0
26	1	47	0.23	0	0	0	441.30	0	24	19	18.3	1	1	70.6	0

**Abbreviations and instructions:** Sex: "1" represents male, "2" represents female. HBsAg^a^: HBsAg at reversion. HBsAg^b^: HBsAg at baseline. HBeAg^a^: HBeAg at baseline. HBeAg^b^: HBeAg status at the time of HBsAg clearance. HBeAg^c^: HBeAg status at reversion. Continue NAs: whether continue NAs after HBsAg reversion. HBV DNA: HBV DNA status at reversion. Presence of CR: Presence of Cirrhosis. HBsAb: HBsAb at stop IFN. Vaccination Status: whether recceived vaccination during treatment. "0" represents negative or no, "1" represents positive or yes. ALT, alanine aminotransferase; AST, aspartate aminotransferase; PLT, platelet; CR, TBIL, total bilirubin; Cirrhosis; NAs, Nucleoside Analogs; Continue NAs: whether continue NAs after HBsAg reversion.

At the time of HBsAg relapse, the average HBsAg level was 0.697 IU/ml, and the HBsAb level was 5.44 IU/ml. During follow-up, the highest HBsAg level observed was 141.84 IU/ml. Among the 26 patients, one lost follow-up and one did not receive treatment, leaving 24 patients who underwent therapy. Of these, 10 received NAs therapy, 3 received PegIFN monotherapy, and 11 received combination therapy with NAs and PegIFN. Eleven of them achieved functional cure, resulting in a functional cure rate of 45.83% (11/24). Furthermore, the recovery rate among patients retreated with Peg-IFN was 78.57% (11/14). The 10 patients in the NA group did not achieve a second cure (detailed information is presented in **Supplementary Table S2**).

## 4 Discussion

Global Burden and Treatment Goals of Chronic Hepatitis B: Chronic hepatitis B (CHB) is a major cause of cirrhosis and hepatocellular carcinoma (HCC) worldwide. Approximately 254 million people are currently infected with hepatitis B virus (HBV), with 1.2 million new infections reported annually. In 2022, HBV-related deaths reached 1.1 million (11). Clinical guidelines highlight that achieving complete virologic control, particularly HBsAg clearance, that is functional cure, in CHB patients significantly reduces the risk of liver decompensation, HCC, and liver-related mortality. These outcomes represent the ideal endpoints for antiviral therapy for CHB (3–5, 7).

Pathways and Challenges in HBsAg Clearance: HBsAg clearance reflects effective immune-mediated suppression of HBV replication, occurring either spontaneously or through antiviral therapy (nucleos(t)ide analogs [NAs] or interferon [IFN]). However, spontaneous and NA-induced HBsAg clearance rates are exceedingly low. In Asian populations, annual rates range from 0.12% to 2.38%, while Western populations exhibit rates of 0.54% to 1.98%. Long-term NA therapy (e.g., entecavir or tenofovir for≥8 years) yields cumulative HBsAg clearance rates of only 1.34%–1.69% (12). STOP-NUC trials demonstrated heterogeneous HBsAg loss rates (2%–10.1%) after treatment discontinuation, whereas PegIFN-based combination therapies significantly enhance clearance rates (40.7%–55.6%) (13–19). Sequential or combined interferon administration may improve HBsAg clearance in select CHB patients, positioning it as a critical strategy for achieving functional cure (19, 20).

Reversion Risks Post-Treatment: Sustained virologic response rates at 1, 2, and 3 years after NA discontinuation are 51.4%, 39.3%, and 38.2%, respectively (13). In patients with compensated cirrhosis, virologic and clinical relapse rates following oral antiviral cessation rise to 55.23% and 43.56% (21). Thus, NAs are often prescribed indefinitely. Conversely, 71.4%–86.63% of patients achieving HBsAg clearance post-PegIFN-α maintained sustained virologic responses (17, 22, 23). Despite adverse effects (e.g., flu-like symptoms, cytopenia), PegIFN-α remains pivotal for functional cure due to its curative potential (2). However, still some patients experienced HBsAg reversion after treatment cessation. Wu et al. (24) reported a cumulative HBsAg reversion rate of 9.66% within a median follow-up of 160 weeks after IFN-induced clearance. Similarly, Li et al. (22) observed a 12.79% reversion rate at 48 weeks post-therapy, while Lok et al, while Lok et al. (25)documented an 18% rate at 96 weeks. These findings likely reflect incomplete eradication of cccDNA or integrated HBV DNA. These findings likely reflect incomplete eradication of cccDNA or integrated HBV DNA in the liver (26). Consistent with these studies, in our cohort, 15.03% (26/173) of patients experienced HBsAg reversion within a median follow-up of 73.3 weeks. Collectively, these data suggested there is still relapse risk and emphasized the importance of long-term monitoring even after HBsAg clearance.

Predictors of HBsAg reversion: Since there is a certain proportion of recurrence rate, identifying predictors of reversion is critical. Elevated baseline HBsAg levels are independent risk factors. High HBsAb titers (>100 mIU/mL) and consolidation therapy exceeding 12 weeks also correlate with sustained functional cure (22, 24, 27–29). Our findings align with prior studies, baseline HBsAg >500 IU/mL (2.7 log) was associated with higher reversion rates (10.40% vs. 4.62%). Patients with HBsAb >100 mIU/mL exhibited lower reversion rates (4.05% vs. 10.98%) and delayed reversion onset (59 vs. 36 weeks, *p<0.05*) (unpublished data). While univariate analysis linked consolidation duration to relapse, this association lost significance in multivariate models, potentially due to limited sample size or confounding by end-of-treatment antibody levels. Larger studies are warranted for validation.

Optimized follow-up intervals explore: Consensus on optimal follow-up intervals post-HBsAg clearance remains limited. Some studies propose 24 weeks as an endpoint for functional cure assessment (30, 31), yet reversion often occurs beyond this window (22, 24). In our cohort, 80.77% (21/26) of reversions emerged within 48 weeks post-PegIFN-α discontinuation, peaking between 24–48 weeks. Thus, intensive monitoring every 12 weeks during the first 48 weeks is advisable. Research indicated patients maintaining HBsAg negative for 1.5 years post-therapy exhibited 96% sustained clearance after 9.6 years (30). In our study, reversion risk neared zero beyond 72 weeks, suggesting this as an ideal endpoint. Extending follow-up to 120 weeks may capture rare late relapses. Reduced intrahepatic cccDNA, HBsAg-positive hepatocytes, and integrated HBV DNA in long-term responders support these recommendations (26, 32).

Safety analysis of post-PegIFN-α HBsAg reversion: Most patients with HBsAg reversion post-PegIFN-α or NA discontinuation remained clinically stable (22, 24, 33). In our cohort, all 26 reversion cases showed normal ALT, AST, and TBIL levels at relapse. Median HBsAg was 0.70 IU/mL (range: 0.05–8.13 IU/mL), with 96.1% (25/26) maintaining undetectable HBV DNA. No cases of liver failure, decompensation, or HCC occurred. Of 24 retreated patients, 45.83% (11/24) achieved secondary HBsAg clearance, all via PegIFN-based regimens, no one came from NAs groups nor without treatment group (**Supplementary Table S2**), consistent with prior reports (34, 35).

Limitations: This study explored the patterns of HBsAg reversion, the predictors of HBsAg reversion, the optimized follow-up intervals in CHB patients who achieved HBsAg clearance through PegIFN-α therapy. These results are crucial for clinicians in monitoring and following these patients. However, there were still some limitations about this study. This was a single-center study, had relatively small sample size, and relatively short follow-up duration. Longer observation and multicenter validation are required. The impact of HBV genotypes on HBsAg clearance durability remains unexplored.

Conclusion: CHB patients attaining HBsAg clearance via PegIFN-α demonstrated favorable long-term outcomes but remain at risk for reversion, predominantly within 48 weeks post-treatment. So we recommend 48 weeks post-treatment to be necessary follow-up endpoint, 72 weeks to be ideal endpoint, and 120 weeks to be extended monitoring endpoint to detect late relapses. This framework provided critical management, though multicenter studies with larger cohorts and longer follow-up are needed to validate findings and explore HBV genotype impacts.

## Data Availability

The original contributions presented in the study are included in the article/**Supplementary Material**. Further inquiries can be directed to the corresponding authors.
