# Supplementary material for: Discussion on the duration of response following HBsAg clearance in patients with chronic hepatitis B treated with PegIFNα-2b
Source: Front Immunol. 2025 Apr 8;16:1518048. doi: 10.3389/fimmu.2025.1518048 (PMC12011802; doi:10.3389/fimmu.2025.1518048)
Supplement: Supplementary file 1 [file Table1.docx]

**Table S1.** Relationship between the time of HBsAg reversion and HBsAb and baseline HBsAg.

| **Time**（weeks） |  | **HBsAb (**IU/ml) | |  | **HBsAg at basline (**IU/ml, lg 500) | |  |
| --- | --- | --- | --- | --- | --- | --- | --- |
|  | N (%) | <100 | ≥100 | *p-value* | <2.7 | ≥2.7 | *p-value* |
| **≤12** | 5 (19.231%) | 5 (26.316%) | 0 (0.000%) | *0.108* | 4 (22.222%) | 1 (12.500%) | *0.79* |
| **>12, ≤24** | 8 (30.769%) | 7 (36.842%) | 1 (14.286%) |  | 6 (33.333%) | 2 (25.000%) |  |
| **>24, ≤48** | 8 (30.769%) | 4 (21.053%) | 4 (57.143%) |  | 5 (27.778%) | 3 (37.500%) |  |
| **>48, ≤72** | 4 (15.385%) | 3 (15.789%) | 1 (14.286%) |  | 2 (11.111%) | 2 (25.000%) |  |
| **>72** | 1 (3.846%) | 0 (0.000%) | 1 (14.286%) |  | 1 (5.556%) | 0 (0.000%) |  |
