# Supplementary material for: Discussion on the duration of response following HBsAg clearance in patients with chronic hepatitis B treated with PegIFNα-2b
Source: Front Immunol. 2025 Apr 8;16:1518048. doi: 10.3389/fimmu.2025.1518048 (PMC12011802; doi:10.3389/fimmu.2025.1518048)
Supplement: Supplementary file 2 [file Table2.docx]

**Table S2.** Follow-Up of Patients with HBsAg Reversion

| No. | HBsAb at HRV（IU/ml） | Treat method after HRV | Highest HBsAg after HRV（IU/ml） | Highest HBVDNA after HRV（IU/ml） | Latest  HBVDNA（IU/ml） | HBsAg turn to negative at latest follow |
| --- | --- | --- | --- | --- | --- | --- |
| 1 | 3.45 | NAs+IFN | 1.88 | 0 | 0 | Negative |
| 2 | 7.85 | NO treatment | 0.46 | 0 | 0 | NO |
| 3 | 0 | NAs+IFN | 0.15 | 0 | 0 | Negative |
| 4 | 7.09 | NAs+IFN | 1.43 | 0 | 0 | Negative |
| 5 | 0 | NAs+IFN | 0.19 | 0 | 0 | Negative |
| 6 | Lost follow | Lost follow | Lost follow | Lost follow | Lost follow | Lost follow |
| 7 | 0 | IFN | 0.49 | 0 | 0 | Negative |
| 8 | 1.47 | NAs | 141.84 | 0 | 0 | NO |
| 9 | 9.23 | NAs+IFN | 2.78 | 0 | 0 | NO |
| 10 | 0.57 | NAs | 0.22 | 0 | 0 | NO |
| 11 | 0 | NAs | 0.09 | 0 | 0 | NO |
| 12 | 11.05 | NAs+IFN | 0.34 | 0 | 0 | NO |
| 13 | 0 | NAs | 63.048 | 1480 | 0 | NO |
| 14 | 2.83 | NAs | 0.17 | 0 | 0 | Negative |
| 15 | 0 | NAs | 57.16 | 0 | 0 | NO |
| 16 | 3.24 | NAs | 1.22 | 0 | 0 | NO |
| 17 | 0 | NAs+IFN | 1.39 | 0 | 0 | Negative |
| 18 | 0 | NAs | 8.13 | 0 | 0 | NO |
| 19 | 0 | IFN | 1.13 | 0 | 0 | Negative |
| 20 | 38.4 | NAs+IFN | 0.34 | 0 | 0 | Negative |
| 21 | 0 | NAs | 0.21 | 0 | 0 | NO |
| 22 | 18.18 | NAs+IFN | 1.36 | 0 | 0 | Negative |
| 23 | 5.35 | NAs | 2.44 | 0 | 0 | NO |
| 24 | 0.72 | NAs+IFN | 0.37 | 0 | 0 | NO |
| 25 | 26.74 | NAs+IFN | 0.25 | 0 | 0 | NO |
| 26 | 0 | NAs | 0.25 | 0 | 0 | Negative |

Abbreviations: IFN, PegIFNa-2b; NA, nucleoside/nucleotide analogues.
